# Stability of the Pregnancy Obsessive-Compulsive Personality Disorder Symptoms Checklist

**DOI:** 10.1007/s00737-017-0793-y

**Published:** 2017-10-24

**Authors:** Kiki E. M. van Broekhoven, Annemiek Karreman, Esther E. Hartman, Victor J. M. Pop

**Affiliations:** Department of Medical and Clinical Psychology, Tilburg University, PO Box 90153, 5000 LE Tilburg, The Netherlands

**Keywords:** Obsessive-compulsive personality disorder, Stability, Personality, Validation

## Abstract

Because stability over time is central to the definition of personality disorder, aim of the current study was to determine the stability of the Pregnancy Obsessive-Compulsive Personality Disorder (OCPD) Symptoms Checklist (*N* = 199 women). Strong positive correlations between assessments at 32 weeks of pregnancy and 2 and 3–3.5 years after childbirth were found (*r* between .62–.72), and the group mean score did not change over time. The Pregnancy OCPD Symptoms Checklist assesses stable, trait-like symptoms of OCPD.

## Introduction

Obsessive-compulsive personality disorder (OCPD) is among the most common personality disorders (PDs) in the general population (3–8%; Diedrich and Voderholzer 2015). Defined by characteristics such as orderliness, perfectionism, and mental and interpersonal control (American Psychiatric Association (APA) 2013), OCPD trait symptoms could interfere with a woman’s adequate adjustment to perinatal changes. During pregnancy, she has to face various kinds of bodily changes, and during labor, contractions resulting in dilation and expulsion occur mostly outside of her control. Also, during the first postpartum years, the young child might have a daily rhythm (feeding and sleeping pattern) and/or temperament that could sometimes be difficult to cope with. Especially women with high levels of OCPD symptoms may encounter (parental) problems during this period. Therefore, although the Pregnancy OCPD Symptoms Checklist is not specific to the perinatal period, we believe that it is important to validate the instrument for use during this specific period. We previously developed the Pregnancy OCPD Symptoms Checklist in a large community sample of pregnant women; a seven-item scale with a one-factor structure proved to be the best fit to the data (Van Broekhoven et al. 2016). A limitation of our previous study is that we had data of only a single assessment at our disposal. Because the stability of PD features over time is crucial to the definition of PDs according to the Diagnostic and Statistical Manual of Mental Disorders (DSM) (APA 2013), studying the stability of OCPD trait symptoms is key to the validation of the Pregnancy OCPD Symptoms Checklist. With repeated assessments of OCPD trait symptoms available to us now, the aim of the current study therefore was to determine the rank-order and mean-level stability of OCPD trait symptoms assessed with the Pregnancy OCPD Symptoms Checklist. Rank-order stability indicates the degree to which the relative ordering of women regarding OCPD trait symptoms is maintained over time, whereas mean-level stability refers to stability of the average trait level of the total group of women (Caspi et al. 2005).

## Materials and methods

This study is part of the Holistic Approach to Pregnancy and the first Postpartum Year (HAPPY) study (for details, see Truijens et al. 2014), a large prospective cohort study of perinatal women. A subsample of women participating in the HAPPY study was subsequently followed in order to extend the assessment of their well-being and provide insight into the development of their children born in the HAPPY study: the HAPPY follow study. Women were asked to complete several questionnaires (received by postal mail or email). Data were collected between March 2013 and July 2017. Baseline characteristics were collected during the first trimester of pregnancy, and OCPD trait symptoms were assessed with the seven-item Pregnancy OCPD Symptoms Checklist at 32 weeks pregnancy and approximately 2 and 3–3.5 years after giving birth (see Appendix; item scores range from 0 to 3 for each item, total scale score ranges from 0 to 21). A total of 199 women who provided data at all three assessments of OCPD trait symptoms were included in the current study. The study was approved by the Medical Ethics Committee of the Máxima Medical Centre Veldhoven, the Netherlands. Informed consent was obtained from all women included in the study.

The previously established one-factor structure of the Pregnancy OCPD Symptoms Checklist was reassessed in the current sample at 32 weeks pregnancy and at 2 and 3–3.5 years after childbirth with principal component exploratory factor analysis (EFA) and confirmatory factor analysis (CFA). Pearson *r* correlations were used to assess rank-order stability of the Pregnancy OCPD Symptoms Checklist score. Mean-level stability was evaluated using repeated measures analysis of variance. Effect size was taken into account for all analyses (Pearson *r* correlation .10 small, .30 medium, .50 large; partial eta squared: .01 small, .06 medium, .14 large; Cohen 1988). Effects that were medium or high were considered clinically relevant. Analyses were performed with the Statistical Package for the Social Sciences (SPSS version 24.0, IBM Corp, Armonk, NY) and AMOS (version 18.0, IBM, Chicago, IL, USA) was used for CFA.

## Results

The mean age of the women was 31 (standard deviation (SD) 3.7) and almost all of them had a partner (98.5%). Half of the women were primiparous, and 95.0% of women had a paid job.

When we again performed an EFA in the current sample of 199 women at 32 weeks pregnancy, we found a two-factor structure with eigenvalues of 3.34 and 1.09, respectively. However, the scree-plot clearly suggested a one-factor solution. Moreover, the second eigenvalue was very low and resulted in a subscale of two items, which is less desirable (at least three items; Pallant 2016). Also, another CFA in this sample using a two-factor structure solution showed a very poor model fit (normed fit index (NFI) of .69, Tucker-Lewis index (TLI) of .57, comparative fit index (CFI) of .71 and a root mean square error of approximation (RMSEA) of .17 with a lower bound of .14; Hu and Bentler 1999). When we used the one-factor solution, a good model fit was found (NFI .94, TLI .95, CFI .96, and a RMSEA .04 with a lower bound of .01) with a total explained variance of 47.8%. For the assessments at 2 and 3–3.5 years postpartum, CFA showed an adequate model fit only for the one-factor solution as well (data not shown). The eigenvalues at 2 and 3–3.5 years after childbirth were 2.66 and 3.05 with a total explained variance of 37.9 and 43.6%, respectively. All item factor loadings were > .40, and Cronbach’s alpha of the checklist ranged from .72–.81 across the three assessments.

### Stability of the pregnancy OCPD symptoms checklist

- Rank-order stability

Pearson *r* correlations between the subsequent assessments of OCPD trait symptoms are shown in Table 1. Total scores on the Pregnancy OCPD Symptoms Checklist were considerably stable over time, with large-sized effects (Pearson *r* correlations .62–.72). Correlation coefficients of different interval lengths (e.g., 32 weeks of pregnancy–2 years after childbirth versus 32 weeks of pregnancy–3–3.5 years after childbirth) were quite similar.

**Table 1:** Correlation matrix of the Pregnancy OCPD Symptoms Checklist scores at subsequent assessments to assess rank-order stability and mean scores and range (*N* = 199)

	32 weeks pregnancy	2 years after childbirth	3–3.5 years after childbirth	Mean (SD)	Range
32 weeks pregnancy	–	.70*	.62*	9.0 (3.7)	1–21
2 years after childbirth	–	–	.72*	9.3 (3.2)	3–19
3–3.5 years after childbirth	–	–	–	9.4 (3.6)	2–20

*OCPD* obsessive-compulsive personality disorder, *SD* standard deviation

*p < .001 (two-tailed)

- Mean-level stability

For the total score on the Pregnancy OCPD Symptoms Checklist, the effect of time was nonsignificant (*F* (1.9, 370.3) = 1.89, *p* = .16, partial eta squared = .009; small effect size), indicating that the group mean score of OCPD trait symptoms remained invariant over time.

## Discussion

The current study showed that OCPD trait symptoms, assessed with the Pregnancy OCPD Symptoms Checklist, were stable over time regarding both relative ordering of the women as well as group means. The fact that the checklist assesses stable and trait-like OCPD symptoms is vital to PD research as PDs are defined as enduring patterns of traits, which are stable over time (APA 2013). Our findings add to the validation and reliability of the Pregnancy OCPD Symptoms Checklist, which has previously been proven to have good psychometric properties (Van Broekhoven et al. 2016). Although the checklist was originally developed for use in pregnant women, its previously found one-factor structure was successfully retested at the later assessments of the current study (2 and 3–3.5 years after childbirth), with all individual items having adequate item factor loadings (i.e., > .40) and satisfactory internal consistency of the total checklist. OCPD symptoms have been reported to be of importance *after* childbirth as well, for example in relation to depression (Akman et al. 2007).

Previous studies have assessed the stability of OCPD features (e.g., Lenzenweger 1999; Shea et al. 2002; Grilo et al. 2004), although none focused on perinatal women. These studies have used a slightly different approach than the current study: whereas we took into account the total score on the Pregnancy OCPD Symptoms Checklist, previous studies primarily focused on the total number of PD features met over time. At the same time, both constitute dimensional approaches. Also, our findings are comparable to those reported in a previous nonclinical sample (Lenzenweger 1999), with relatively high rank-order stability of OCPD features across a four-year follow-up period (.40–.66). For only one of two instruments used in this study, a significant—but small—decrease in the mean number of OCPD features met over time was reported (Lenzenweger 1999). A clinical population study of the stability of OCPD features with multiple assessments over a one-year period reported notably higher rank-order stability (.86–.90) (Shea et al. 2002), which is possibly attributable to the fact that this clinical study had a broader range of severity in PD scores (Shea et al. 2002) and a shorter period of follow-up compared to the current study. A clinical population study with repeated assessments over a two-year period, however, reported rank-order stability very similar to ours (.53–.79) (Grilo et al. 2004). In addition, clinical studies tend to report a decrease in mean number of OCPD criteria met over time (Shea et al. 2002; Grilo et al. 2004). Our relatively high rank-order stability is comparable to that found for major normal personality dimensions (test-retest correlation of .62 between the ages of 30–39; Roberts and DelVecchio 2000).

A strength of the current study is that we assessed OCPD trait symptoms at three time points, which enabled us to compare stability coefficients across different interval lengths. Rank-order stability proved to be rather similar across different interval lengths, which is important to the definition of stability of a construct (Fraley and Roberts 2005). The fact that the Pregnancy OCPD Symptoms Checklist does not cover all DSM-5 criteria OCPD criteria (APA 2013) could be considered a limitation of our study. However, components of the defining features of OCPD (APA 2013) are covered by the checklist. The checklist covers need for control and the DSM-5 OCPD criteria of perfectionism, preoccupation with details, and excessive devotion to work and productivity. The criteria of rigidity and stubbornness, reluctance to delegate or work with others, inability to discard worn-out or worthless objects, miserliness, and overconscientiousness are not covered by the questionnaire. In addition, our interest was in the stability of *a subset of* OCPD trait symptoms assessed with the Pregnancy OCPD Symptoms Checklist, as it was previously developed in a nonclinical, community sample of pregnant women (Van Broekhoven et al. 2016).

In conclusion, the Pregnancy OCPD Symptoms Checklist assesses stable, trait-like symptoms of OCPD, which is central to the generic definition of DSM PDs (APA 2013). OCPD has hardly been addressed in perinatal mental health research, while its comorbidity with affective disorders in the general population is 24% (Diedrich and Voderholzer 2015). Moreover, the perinatal period is characterized by events that might be difficult to cope with for women with OCPD symptoms. As such, our checklist could be a valuable screening tool to identify women during pregnancy who are at risk for perinatal depression.

## Appendix

**ᅟ:** The Pregnancy OCPD Symptoms Checklist. Below, you will find several statements that could describe general characteristics. Please indicate to what extent each of these statements could apply to you *in general*

	Never	Sometimes	Often	Always
1. I like to keep a clear overview of the things I am doing or I am someone who is generally organized, orderly, and detail-oriented.	O	O	O	O
2. I am the type of person who memorizes or writes down various lists and planning schemes.	O	O	O	O
3. I find it difficult to finish work because I spend a lot of time doing everything as well as possible.	O	O	O	O
4. Often I am so busy with things that need to be done that I do not allow myself time for pleasure or relaxation.	O	O	O	O
5. I want to be in control of everything and have a hard time when something unexpected intervenes.	O	O	O	O
6. I become restless and panicky when I feel like I am not in control of everything.	O	O	O	O
7. I feel useless and worthless when I do not experience control over my own life.	O	O	O	O

Item scores range from 0 to 3; total scale score ranges from 0 to 21

*OCPD* obsessive-compulsive personality disorder
